# 264 Building Empowerment through FITness (BeFIT) – CORRIGENDUM

**DOI:** 10.1017/cts.2024.633

**Published:** 2024-10-22

**Authors:** Kimberly McCall, Keith McGregor, Jewell Dickson, Raymond Jones, Samantha Giordano-Mooga, Shellie Layne

The above abstract published with an error in the author list. The correct list is shown below.

Kimberly McCall, PhD, Assistant Professor; Keith McGregor, PhD, Associate Professor; Jewell Dickson, OTD, Associate Professor; Raymond Jones, PhD, Assistant Professor; Samantha Giordano-Mooga, Assistant Professor, PhD; and Shellie Layne, M.Ed. Women Under Construction Network/CEO
